# Linear Multiplication Beyond Geiger Mode Threshold in Ge-on-Si Avalanche Photodiode

**DOI:** 10.3390/mi17060726

**Published:** 2026-06-15

**Authors:** Dongyan Zhao, Qiang Wen, Fang Liu, Wei Qi, Sichao Du

**Affiliations:** 1 Beijing Smart-Chip Microelectronics Technology Company Ltd., Beijing 100192, China; liufang@sgchip.sgcc.com.cn; 2 College of Integrated Circuits, Zhejiang University (ZJU), Hangzhou 311200, China; 3 Zhejiang Key Laboratory of Quantum Materials and Control, Zhejiang Engineering Research Center for Edge Intelligence Technologies and Equipment, School of Information and Electrical Engineering, Hangzhou City University, Hangzhou 310015, China; 2240201029@stu.hzcu.edu.cn (Q.W.); qiw@hzcu.edu.cn (W.Q.); 4 Zhejiang Province Key Laboratory of Intelligent Electromagnetic Control and Electronic Integration, Innovative Institute of Electromagnetic Information and Electronic Integration, College of Information Science and Electronic Engineering, Zhejiang University (ZJU), Hangzhou 310027, China

**Keywords:** avalanche photodiode, Ge-on-Si, Geiger mode, interface traps, linear multiplication, short-wave infrared detection

## Abstract

This research investigates a vertically structured Ge-on-Si avalanche photodetector (APD) fabricated in a separate absorption, charge, and multiplication configuration. The application of ramp gating enables reverse bias beyond the punch-through voltage, allowing the device to operate in linear avalanche mode. A significant dark avalanche current is observed under steady conditions, exhibiting linear multiplication approximately proportional to the input gating and thermal generation rate. Notably, this linear behavior persists even at voltages beyond the Geiger mode. The observed results are attributed to Ge/Si interface traps caused by the 4.18% lattice mismatch and deep-level traps introduced during fabrication. Under 1550 nm short-wave infrared normal-incidence pulsed illumination, the device exhibits negative differential resistance, attributed to illumination-induced self-quenching of electric field in multiplication region and modification of the barrier at the Ge/Si interface. A light-induced slow transient decrease in the absolute dark-state current is followed by a sustained inverse quenching effect, restoring the large dark-state current. These findings offer insights into the dynamic behavior of Ge-on-Si APDs, with potential implications for advanced optoelectronic applications.

## 1. Introduction

Germanium-on-silicon (Ge-on-Si) photodetectors (PDs) play a vital role in silicon photonics applications, including sensing, communication, and optical interconnections. They are particularly valued for their affordability and compatibility with complementary metal-oxide-semiconductor (CMOS) technology, which facilitates their integration into contemporary electronic systems [1,2,3,4,5]. The seamless integration of Ge-on-Si photodetectors with CMOS processes renders them indispensable for the development of photonics-based application-specific integrated circuits (ASICs). The practical viability of this technology in high-end applications is evidenced by the successful deployment of monolithic photonic modules by major industry entities such as Intel and IBM [6,7,8]. Recently, the application of Ge-on-Si photodetectors has rapidly expanded into the realm of ultra-sensitive single-photon detection for short-wave infrared (SWIR) light detection and ranging (LiDAR) and 3D imaging [9,10].

The 4.18% lattice mismatch between Ge and Si remains a major obstacle for these photodetectors, despite their other advantages. This mismatch leads to a high density of dislocations at the Ge/Si interface and results in threading dislocations within the Ge epitaxial layers [11,12,13]. Initially, the silicon layer served primarily as a substrate, with PDs being fabricated entirely from Ge material to bypass the issues associated with the interface [14]. However, recent breakthroughs in epitaxial growth techniques have permitted the fabrication of Ge-on-Si heterojunction devices, wherein the silicon substrate also functions effectively as an electrical contact. This development has sparked significant research and widespread adoption of these devices. Other interfaces in the device also possess specific trap densities, with deep traps acting as charge storage and surface traps affecting response time and sensitivity. It is observed that various device interfaces exhibit distinct trapping mechanisms; specifically, deep-level defects contribute to charge retention, while surface states modulate the device’s transient response and sensitivity. Although fine-tuning these trap profiles offers a pathway to enhance responsivity and enable novel device concepts, rigorous control is required, as an excessive density of defects inevitably degrades efficiency [15,16,17,18,19,20,21,22,23].

Within the realm of high-speed detection, the vertical p+(Ge)-i(Ge)-n+(Si) architecture has emerged as a dominant design [24,25,26,27]. However, its performance is often compromised by severe lattice mismatch, which introduces a high density of interfacial dislocations. These defects significantly amplify the generation-recombination current via trap-assisted tunneling [28]. Furthermore, when combined with the bulk SRH processes typical of narrow-bandgap materials, the resulting dark current density reaches approximately 44.1 mA/cm^2^, a value orders of magnitude higher than the 15.2 µA/cm^2^ observed in mature III–V counterparts [29]. To diagnose these leakage pathways in both standard and SOI-based devices, extraction of activation energies and ideality factors is typically required [30,31].

When the reverse bias across an APD increases and reaches the punch-through voltage, the dark current rises sharply. Beyond this point, the APD operates in linear multiplication mode. Further increasing the reverse bias past the avalanche threshold voltage transitions the device into Geiger mode [32,33,34,35,36]. The APD’s performance depends on its design and the accompanying detection circuitry. Quenching circuitry is crucial to rapidly suppress the avalanche current by reducing the reverse bias across the APD [37]. During an avalanche event, the photo voltage across the readout element temporarily rises, lowering the electric field in the multiplication region. This voltage then decreases via quenching until the carrier multiplication threshold is restored.

This research explores a vertically structured Ge-on-Si avalanche photodetector with separate absorption, charge, and multiplication layers. The device’s current–voltage behavior is shaped by carrier trapping and annihilation due to lattice mismatch and fabrication-induced defects. It operates in linear avalanche mode even beyond Geiger voltage, showing stable dark avalanche current. Under 1550 nm infrared illumination, it displays negative differential resistance and unique transient current effects. These findings provide valuable insights for advanced optoelectronic applications.

## 2. Device Fabrication

**Figure 1:**
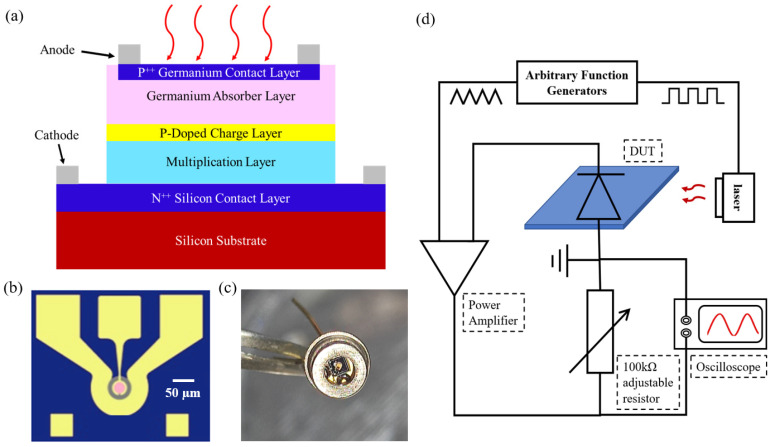
(**a**) An illustration showing various sequentially, and epitaxial grown layers of the vertical Ge-on-Si PD. (**b**) A high-resolution top-view layout schematic of the employed APD is incorporated. (**c**) The actual vertical Ge-on-Si APD in ready-to-test packaged form. (**d**) Schematic diagram of the transient optical response test system for APD devices.

The fabricated Ge-on-Si APD is schematically illustrated in Figure 1a. The device design employs a vertical separate absorption, charge, and multiplication (SACM) configuration. A 100 nm thick N^++^ silicon contact layer (1.0 × 10^20^ cm^−3^) is grown on a 200 µm thick silicon substrate. This is followed by the sequential growth of the silicon-based multiplication layer (1.0 × 10^15^ cm^−3^) characterized as *W*_m_ = 1000 nm. Then, a 100 nm thick p-doped silicon film (1.0 × 10^17^ cm^−3^) acting as the charge layer is deposited. This is followed by a 1000-nm-thick Ge absorber layer (1.0 × 10^15^ cm^−3^), whose absorption spectrum enables it to photo-ionize under 1550 nm wavelength. Finally, a 100 nm thick P++ Ge contact layer (1.0 × 10^20^ cm^−3^) is grown onto the absorber layer, followed by standard photolithography and etching processes to define the mesa structure for the top contact. Figure 1b depicts the high-resolution top-view layout schematic of the Ge-on-Si photodetector. Meanwhile, Figure 1c displays a real image of the ready-to-test package containing the APD. As shown in Figure 1d the main test equipment employed in this system includes an arbitrary function generator, a power amplifier, a pulsed laser, and an oscilloscope. The arbitrary function generator drives the pulsed laser, while the power amplifier applies a ramped bias voltage to the APD device. The APD current signal is converted into a voltage signal through a series load resistor and is ultimately acquired in real time by the oscilloscope.

## 3. Results and Discussion

**Figure 2:**
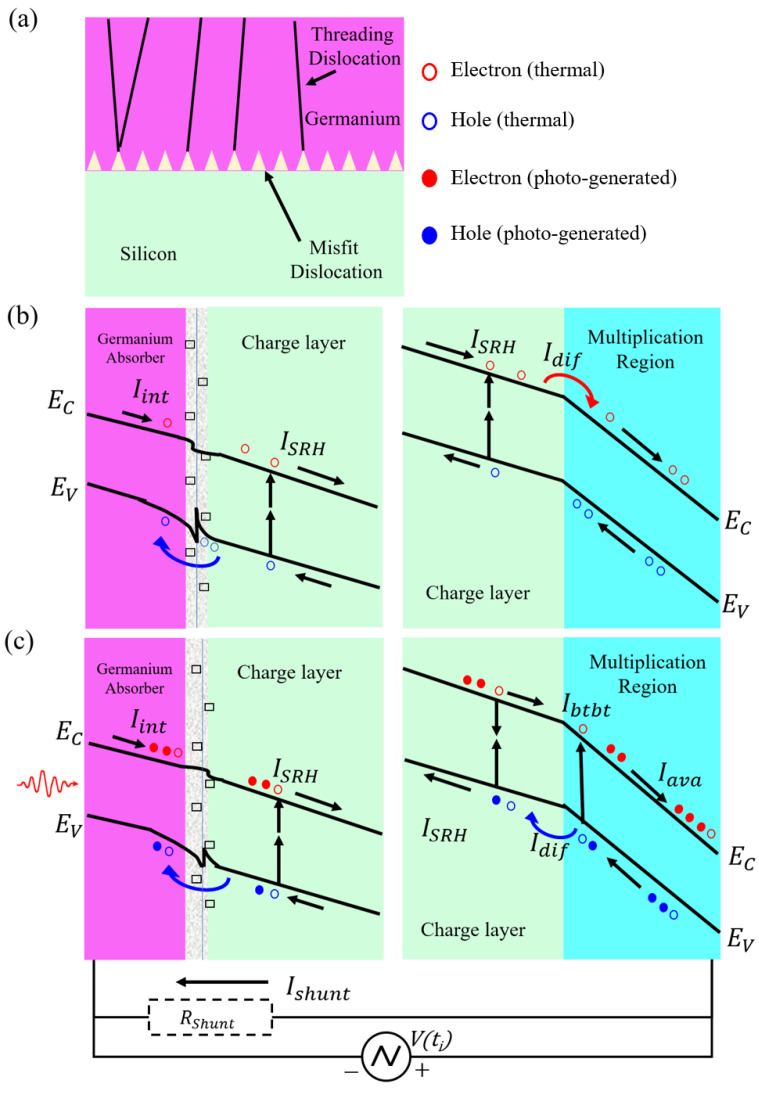
The schematic diagram illustrating the dislocation distribution at Ge/Si interface is shown in (**a**). Band diagrams of the reverse biased Ge-on-Si APD under (**b**) dark and (**c**) illumination conditions are shown.

Utilizing low-cost and readily accessible silicon wafers as substrates for Ge epitaxy offers a financially viable pathway for device integration, a strategy highlighted in this study. Such Ge-on-Si architectures are vital not only for improving the spectral response of silicon but also for facilitating the simultaneous fabrication of ancillary silicon hardware, including biosensing detectors and image sensors. Despite these clear benefits, the physical combination of Ge and Si is severely hindered by their crystallographic lattice mismatch. The difficulty of this integration is further compounded by the fact that these materials exhibit distinct thermal expansion behaviors. Lattice mismatch is mathematically expressed in as

(1) $$\Delta \alpha /\alpha =(\alpha_{e}-\alpha_{s})/\alpha_{s}=(\alpha_{\mathrm{Ge}}-\alpha_{\mathrm{Si}})/\alpha_{\mathrm{Si}}=4.18\%$$

where $\alpha_{e}$ represents the lattice parameter of the epitaxial layer, and $\alpha_{s}$ refers to that of the substrate material. The resulting lattice mismatch induces a substantial accumulation of strain energy within the Ge layers that are epitaxially grown on Si. This strain energy is primarily alleviated through two mechanisms: (i) the formation of lattice dislocations at the interface (known as misfit dislocations) and (ii) the elastic deformation of both the substrate and the Ge islands that develop on the surface during the initial stages of growth, following the Stranski–Krastanow growth mode [38]. Typically, elastic deformation is insufficient to fully relieve the accumulated strain, which results in the nucleation of misfit dislocations at the periphery of the Ge islands [39]. Frequently, these dislocations curve towards the growth direction and propagate upward, appearing as threading dislocations at the surface, as depicted in Figure 2a. The presence of these defects is severely detrimental to charge transport performance, as they degrade carrier mobility and limit carrier lifetimes. Therefore, it is essential to implement growth strategies that can effectively bridge the lattice constant gap between the two materials. For a specific PD, the current flowing through it can be expressed as *I* = *I*_dark_ + *I*_pc_, where *I*_dark_ represents the dark current, and *I*_pc_ denotes the current component attributed to photogenerated carriers.

The composition of *I*_dark_, as detailed in Figure 2b,c, encompasses six fundamental mechanisms. These are constituted by the bulk-related diffusion (*I*_dif_) and SRH generation-recombination currents (*I*_SRH_), alongside the interface-mediated trap-assisted tunneling (*I*_int_). Additionally, the device exhibits field-dependent components, specifically band-to-band tunneling (*I*_btbt_) and avalanche current (*I*_ava_), as well as leakage attributed to the shunt resistance (*I*_shunt_). The overall dark current can be expressed as

(2) $$I_{\mathrm{dark}}=I_{\mathrm{dif}}+I_{\mathrm{SRH}}+I_{\mathrm{int}}+I_{\mathrm{btbt}}+I_{\mathrm{ava}}+I_{\mathrm{shunt}}$$

It is important to note that *I*_btbt_ and *I*_ava_ are generated at electric fields greater than > 3 × 10^5^ V/cm. These two current components exhibit negligible effects under low-bias testing conditions. *I*_shunt_ is defined as *I*_shunt_ = *V*/*R*_s_, where the shunt resistance (*R*_s_) is typically in an order of 10^11^ Ω, rendering *I*_shunt_ negligible in this context [40]. Moreover, the principle of conservation in dissipative transition energy suggests that the rate of electron capture into neutral traps is greater for trap energy levels that are closer to the conduction band edge [41].

In this section, we systematically evaluate the device’s photoelectric conversion capability and avalanche multiplication characteristics under high bias. All optical measurements were performed at room temperature using an unmodulated laser with a wavelength of 1550 nm as the light source, and the incident optical power was fixed at 100 µW. A unique ramp gate voltage sweeping technique was employed during the measurement, as shown in Figure S1a,b (Supporting Information). Within a scanning period of 0.1 s, the applied reverse bias decreases linearly from the maximum peak voltage (−25 V to −50 V) to 0 V, and then increases linearly back to the maximum peak voltage. We extracted the corresponding current and voltage parameters. For the Ge-on-Si avalanche photodiode with a silicon multiplication layer thickness Wm = 1 µm, the punch-through voltage is reduced to approximately −0.15 V to −0.25 V.

**Figure 3:**
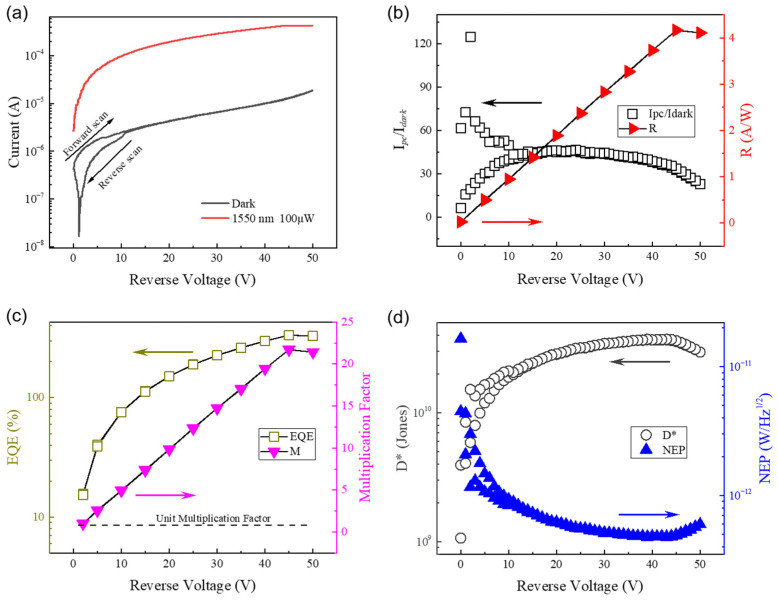
(**a**) Dark current and photocurrent characteristics of the Ge-on-Si APD under 100 µW unmodulated illumination; (**b**) Photocurrent-to-dark-current ratio (*I*_pc_/*I*_dark_) and responsivity (*R*) characteristics; (**c**) Dependence of external quantum efficiency (*EQE*) and avalanche multiplication gain on bias voltage; (**d**) Specific detectivity (D*) and equivalent noise power (*NEP*) characteristics.

Figure 3a illustrates the dark current and photocurrent characteristics of the device over a reverse bias range from 0 V to 50 V. The I–V curve sweep was intentionally terminated at −50 V due to equipment limitations, to prevent device damage caused by continuous Joule heating. The dark current curve exhibits a distinct hysteresis loop between the forward sweep and the reverse sweep. This hysteresis phenomenon in heteroepitaxial devices is typically associated with defect states or deep-level traps at the material interface. Due to the approximately 4.2% lattice mismatch between Ge and Si, threading dislocations and interface states are inevitably introduced during the epitaxial growth process. Under high reverse bias, these defect centers capture carriers; when the voltage decreases, the trapped carriers are gradually released, causing deviations in the dark current value.

We extracted the curves of the photocurrent-to-dark-current ratio (*I*_pc_/*I*_dark_) and responsivity (*R*) as a function of reverse sweep bias voltage, as shown in Figure 3b. With the increase in reverse bias voltage, the responsivity R of the device exhibits a monotonically increasing trend. This indicates that as the electric field strength rises, the collection efficiency of primary photogenerated carriers is continuously improved, and the avalanche multiplication effect is triggered in the Si multiplication layer at higher bias voltages. When the bias voltage reaches approximately 45 V, the responsivity hits a maximum of 4.16 A/W due to intense impact ionization. However, as the bias voltage increases further, the responsivity curve shows a trend of saturation followed by a slight decrease, which is mainly attributed to the space charge screening effect induced by the accumulation of high-concentration carriers in the multiplication region.

It is worth noting that a characteristic peak of the photocurrent-to-dark-current ratio appears in the low bias voltage region. This is mainly because under near-zero bias or weak reverse bias conditions, the intrinsic dark current of the device is greatly suppressed and approaches the noise floor of the test system; meanwhile, the photogenerated carriers in the top Ge absorption layer have begun to be effectively swept out by the built-in electric field, generating a considerable photocurrent and thus forming a high transient ratio. In the moderate bias voltage range from 10 V to 40 V, *I*_pc_/*I*_dark_ is basically stabilized at a high level of around 45, indicating that the device has excellent linear response at this stage. However, when the bias voltage exceeds 40 V and enters the deep avalanche multiplication region, *I*_pc_/*I*_dark_ begins to show an obvious saturation trend. This is mainly because in the deep avalanche region, the defect energy levels at the Ge/Si heteroepitaxial interface are activated, and the bulk dark current (especially the defect-assisted generation-recombination current) participates in the multiplication process, with its exponential growth rate exceeding that of the photocurrent.

The dependence of the external quantum efficiency (*EQE*) and avalanche multiplication factor (*M*) of the device on the reverse sweep bias voltage is presented in Figure 3c. In the low bias voltage region, the primary photogenerated carriers are swept out by the built-in electric field, the collection efficiency of photogenerated carriers is gradually enhanced, and the *EQE* shows a rapid upward trend. At a reverse bias voltage of approximately 2 V, the current shows a pronounced upward trend with increasing voltage. The distinct inflection point in the first derivative of dark current with respect to reverse bias voltage here indicates that the electric field within the silicon multiplication layer has reached the threshold of impact ionization, with the multiplication factor of the device approaching 1 at this moment. The multiplication factor (*M*) is defined as the ratio of the net photocurrent at a specific voltage to the primary net photocurrent, given by $M=(I_{pc}-I_{dark})/(I_{pc(ref)}-I_{dark(ref)})$. With the further increase in the reverse bias voltage, the additional electric field mainly acts on the silicon (Si) multiplication layer. An intense impact ionization process is triggered inside the Si multiplication layer, where avalanche multiplication of photogenerated carriers occurs. At this point, the *EQE* exceeds 100% and shows a linear increasing trend together with the multiplication gain *M*. When the bias voltage is close to 45 V, the multiplication gain of the device reaches the maximum value of *M* ≈ 21.7, with the corresponding *EQE* exceeding 330%. However, as the bias voltage further approaches 50 V, the gain *M* and *EQE* show a slight trend of decrease and saturation. This is mainly because under the high-gain state, high-concentration photogenerated carriers accumulate in the multiplication region, which induces a space charge effect, weakens the local effective electric field, and thus suppresses the avalanche multiplication capability of the device.

Regarding the evaluation of the weak light detection limit, *NEP* is a metric indicating the optical power required for a signal-to-noise ratio of 1. Assuming the device noise is dominated by shot noise, the *NEP* is calculated as $NEP=\sqrt{2qI_{dark}}/R$, where q is the elementary charge, *I*_dark_ is the total measured dark current, and R is the responsivity. As shown in Figure 3d, benefiting from the effective amplification of the optical signal by avalanche gain, the device’s *NEP* decreases rapidly from the background-noise-dominated region at low voltages, reaching as low as approximately 4.4 × 10^−13^ $W/\sqrt{Hz}$ at a bias of 45 V. Its specific detectivity (D*) is calculated using the formula $D^{*}=R\sqrt{A}/\sqrt{2eI_{dark}}$, where A is the effective active area. Specific detectivity is helpful for comparing the photodetection-related figures of merit of devices with different active areas under the same *I*_dark_. Ultimately, thanks to the effective blocking of intrinsic dark current by the SACM heterostructure and the effect of avalanche multiplication under dynamic bias, the specific detectivity of the device reaches a maximum of approximately 4 × 10^10^ Jones near the peak bias of 45 V. Beyond 45 V, D* begins to decrease and *NEP* rebounds, further confirming that excessively high bias leads to a sharp increase in multiplied dark current noise and space charge saturation effects, thereby deteriorating the overall signal-to-noise ratio of the device.

We also tested the transient photoresponse characteristics of the germanium-on-silicon avalanche photodiodes (Ge-on-Si APDs), with the test system shown in Figure 1d. The APD device is connected in series with a 100 kΩ load resistor (*R*_L_), which functions as a transimpedance amplifier in the circuit, converting the current signal from the APD into a voltage signal captured by the oscilloscope. A ramp gating signal is generated by the arbitrary function generator and amplified by a power amplifier; the ramp gating voltage applied to the device linearly sweeps from −10 V to −80 V over a duration of 1 ms. Simultaneously, the arbitrary function generator drives a pulsed laser with a wavelength of 1550 nm, a pulse width of 10 ns, a laser power of 60 μW, and a repetition rate of 1 kHz. The oscilloscope records the voltage changes across the load resistor in real time, thereby obtaining the *I*–*T* curves of the dark current and photocurrent over time.

**Figure 4:**
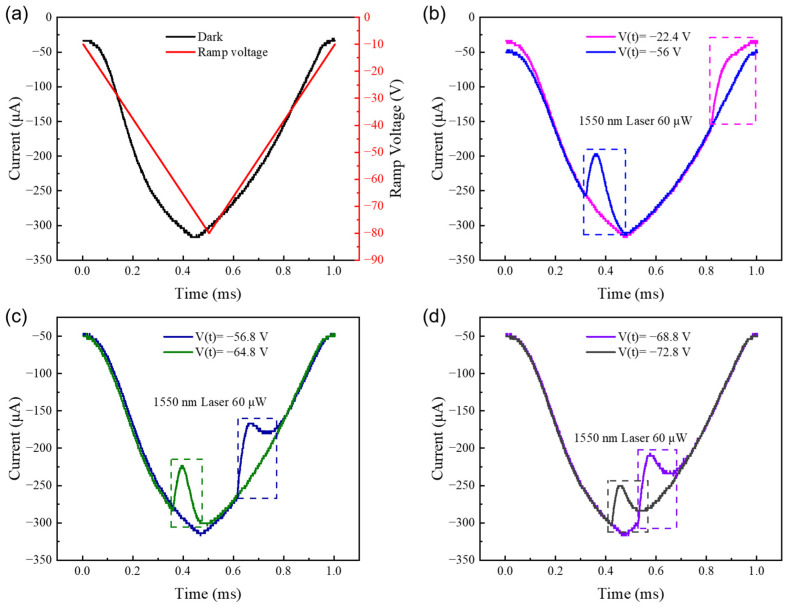
Transient photoresponse characteristics of the device under ramp gate voltage. (**a**) Dark current curve as a function of the ramp gate voltage, and the time-dependent profile of the voltage applied across the device; (**b**–**d**) Photoresponse curves triggered by turning on the 1550 nm laser (optical power: 60 μW) at different instantaneous voltages *V*(*t_i_*) during the application of the same ramp bias voltage. The dashed boxes in the figures denote the characteristics of the transient photocurrent response of the device at a specific *V*(*t_i_*) at the moment of laser turn-on.

In Figure 4, *I*–*T* curves for the temporal variation of dark- and photo-current under normal incidence of 1550 nm wavelength pulsed laser are plotted over a single cycle of input ramp gating *V* which varies between −10 V to −80 V. When the laser pulse irradiates the device, the corresponding instantaneous gating voltages *V*(*t_i_*) are synchronously recorded. This helps exploit varying electric fields inside the multiplication region. All of the photo-ignited impact ionization events are plotted simultaneously to understand the transient photo response of the Ge-on-Si APD for fixed pulsed illumination.

As shown in Figure 4a, the dark current over the entire gating cycle is notably large. The possible sources of such large dark current include the leakage requiring fabrication optimization, field enhanced tunneling, high-field initiated thermal generation rate in Si, and excessive thermal generation in Ge absorber due to the absence of cryogenic device testing conditions. Traditionally, as the reverse bias on an APD is increased and the punch-through voltage is reached, the device enters linear avalanche operation—carrier multiplication then scales linearly with both the incident power density and the applied bias. When an APD exceeds its breakdown voltage, it enters Geiger mode and experiences uncontrolled avalanche breakdown. However, during the ramp gating cycle, this device did not exhibit the typical runaway current behavior characteristic of traditional Geiger mode. Instead, it demonstrated a stable dark avalanche current that increased approximately linearly with voltage.

In Figure 4b (*V*(*t*) = −22.4V), under a relatively low instantaneous gating voltage, the short-term photogenerated component induced by the optical pulse is weak, followed by a slight and slow current decay. This behavior can be attributed to the capture of photogenerated carriers at interface traps, which partially screen the electric field in the multiplication region over a short period, thereby suppressing the output current. This reflects the trapping effect of defects, though the overall field strength remains insufficient to amplify this phenomenon. Figure 4b–d reveal that as the instantaneous gating voltage increases, both the transient amplitude and slow decay become more pronounced. This indicates enhanced tunneling or field-enhanced recombination processes involving traps under higher electric field conditions, demonstrating that the capture-delayed emission dynamics of traps exert more significant modulation on the current. Under high electric fields, these defects frequently trap and release carriers, thereby restricting the mean free path of the carriers and the probability of impact ionization. This creates a “self-limiting” effect that suppresses avalanche runaway, allowing the device to maintain a linear response even in Geiger mode. The practical advantage is that it provides inherent protection against catastrophic breakdown without the need for complex external quenching circuits. This characteristic greatly simplifies the readout circuit in high-gain application scenarios.

Under 1550 nm pulsed illumination, the device exhibits negative differential resistance (NDR) effect and inverse quenching phenomenon. This NDR behavior is primarily driven by the space-charge effect. Similar space-charge screening and self-quenching mechanisms, which dynamically distort the local electric field to suppress avalanche multiplication, have been broadly investigated in various APDs, including high-speed Ge-on-Si structures [42,43]. Specifically, the massive injection and subsequent separation of photogenerated electrons and holes in the multiplication region generate a counter-built-in electric field. This localized screening effect instantaneously reduces the effective electric field strength and avalanche gain, which macroscopically manifests as a current decrease under illumination. Additionally, this self-quenching process is accompanied by illumination-induced reconstruction of the charge distribution at the Ge/Si interface, which further modifies the interface potential barrier.

A first-order exponential fit to the transient current recovery edge yields effective carrier release time constants that decrease from approximately 78 μs to 46 μs as the reverse bias increases. This originates from the introduction of a high density of threading dislocations at the Ge/Si interface [44], while the high electric field significantly accelerates the thermal emission rate of these deep traps through the Poole–Frenkel effect and trap-assisted tunneling [45,46,47].

Concurrently, a significant current hysteresis is observed during the dynamic voltage sweeps, with the current difference between forward and reverse sweeps reaching up to 50 μA at larger bias voltages. For the Ge-on-Si heterostructure, this hysteresis is jointly attributed to three dominant physical mechanisms: (i) charge trapping at Ge/Si interfacial defects, (ii) deep-level trap states within the depletion region, and (iii) non-abrupt junction profiles caused by dopant interdiffusion during thermal processing. This trap-driven modulation aligns with recent studies on diverse semiconductor heterojunctions, demonstrating that carrier trapping/detrapping dynamics at interfacial defects and trap-assisted tunneling are fundamentally responsible for inducing both NDR and prominent hysteresis behaviors [48,49,50,51]. Under high electric fields, the capture-delay-emission dynamics of these deep-level traps actively participate in field-assisted recombination and tunneling processes. Ultimately, the combined effects of the charge storage within these traps and the smeared junction profile dictate the pronounced hysteresis and strongly modulate the macroscopic transient current.

The temporal noise sources for the Ge-on-Si APD can be characterized into three main parts [52]: photon shot noise, dark-current shot noise, and readout noise. Both photon and dark-current shot noise originate from the intrinsic statistical nature of carrier generation processes in the absorber layer. In contrast, the readout noise arises from charge-to-voltage conversion and the successive processing.

**Figure 5:**
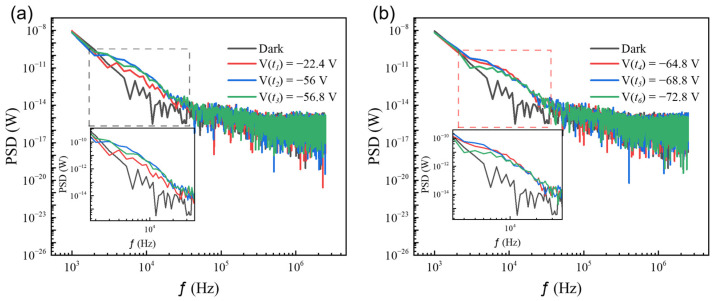
Power spectral density (PSD) calculated over the complete ramp gating cycle. (**a**) PSD spectra obtained in the dark state and under 1550 nm pulsed-laser illumination with corresponding *V*(*t_i_*) values of −22.4, −56.0, and −56.8 V. (**b**) PSD spectra obtained in the dark state and under 1550 nm pulsed-laser illumination with corresponding *V*(*t_i_*) values of −64.8, −68.8, and −72.8 V.

To analyze the sources and proportional contributions of these noise components, we performed Fast Fourier Transform (FFT) on the transient current (*I*–*T*) signals presented in Figure 4, and extracted the Power Spectral Density (PSD) of the transient photoresponse under different bias voltages (V(*t_i_*)), as shown in Figure 5. As can be seen from the spectrum plot, the PSD curves exhibit two distinct frequency-domain characteristics: in the low-frequency region (below 10^5^ Hz), the PSD shows an obvious frequency dependence (1/f noise). As illustrated by the magnified details in the inset, the noise power spectral density corresponding to the transient photoresponse under high applied reverse bias is significantly increased compared with that in the dark state (Dark). This is mainly attributed to the fact that the intrinsic shot noise is amplified by thermal fluctuations, resistance variations, and the carrier multiplication process inside the device under a strong electric field. In contrast, in the high-frequency region (above 10^5^ Hz), the PSD curves under different bias voltages gradually flatten out and converge, exhibiting typical white noise characteristics. This bias-independent flat noise floor indicates that the primary noise source of the device in the high-frequency band is no longer the carrier-related physical processes within the APD, but is dominated by the external readout circuit, including the charge-to-current conversion process of the transimpedance amplifier and the intrinsic system noise floor of the oscilloscope.

Finally, Table 1 compares the performance of our vertical SACM Ge-on-Si APD with recently reported Ge-on-Si and III-V avalanche photodetectors. Here, Δ*V_linear_* denotes the operating voltage range over which linear avalanche multiplication is sustained, i.e., the voltage range from the onset of impact ionization (avalanche effect) to the onset of current runaway.

Although a full system-level demonstration is slated for future work, the practical implications of this novel operational regime offer distinct theoretical advantages over conventional Geiger-mode operation. In conventional single-photon avalanche diodes (SPADs), once an avalanche is triggered, complex quenching circuits must be employed. These components occupy a significant physical area, severely limiting the fill factor of large-scale detector arrays. In contrast, the space-charge-induced negative differential resistance (NDR) phenomenon in our device serves as a protection mechanism, autonomously lowering the electric field in the multiplication region under high bias or high optical intensity, and holding promise for higher-density pixel integration in silicon photonic imaging systems. Furthermore, a key practical implication of our device is its intrinsic photon number resolving (PNR) capability based on the linear Geiger mode. Conventional SPADs only output binary values (0 or 1) and require complex array structures such as silicon photomultipliers (SiPMs) to distinguish between single-photon and multi-photon incidence. Specifically, linear avalanche multiplication maintains stability in high-field regions without triggering the current runaway characteristic of Geiger mode, thereby extending the operational range of the device. The output current remains proportional to the incident optical power, enabling photon number resolving, and this feature consequently simplifies the design of the external readout circuitry.

Deep and surface traps in Ge-on-Si APDs can be strategically engineered to enhance device performance for specialized applications. By tailoring trap characteristics, it is possible to improve responsivity, reduce noise, and develop innovative device architectures. However, careful engineering is essential to prevent inefficiencies arising from excessive trap densities. Advanced optimization techniques include trap engineering, surface passivation strategies, temperature control mechanisms, and optimized device geometries, all supported by real-time feedback mechanisms [60,61]. Consequently, these cost-efficient detectors offer seamless compatibility with silicon photonics, making them indispensable for high-speed optical data transmission within the standard telecommunication wavelengths. Beyond optical communications, their versatility meets the stringent demands of emerging technologies, ranging from free-space sensing and adaptive optics to LiDAR systems for autonomous driving and ultra-weak light detection.

## 4. Conclusions

For the employed Ge-on-Si APD, a notable dark avalanche current is observed under steady-state conditions, exhibiting linear multiplication proportional to both the applied gating and thermal generation rate. This linear behavior persists even at voltages exceeding the Geiger mode threshold. The results are attributed to Ge/Si interface traps caused by the 4.18% lattice mismatch and deep-level traps introduced during fabrication. The associated negative differential resistance is correlated with the photoinduced self-quenching of lateral electric fields within symmetrically configured multiplication regions and modifications to the potential barrier at the Ge/Si interface.

## Figures and Tables

**Table 1:** Comparison of the Devices with Various Photodetectors.

Device Structure	*M*	*R*(*A*/*W*)	*I*_dark_	*V_br_*(V)	ΔV*_Linear_*	NDR	λ(μm)	Ref
Lateral SAC Ge-on-Si APD	30	14.8	40.7 nA	5.81	2.31	N/A	1550	[53]
Ge/Si APD	39	12	5 μA	29.4	18	N/A	1550	[54]
InGaAs SACM APD	22	10.8	19 μA/cm^2^	22	9	N/A	1310	[55]
Vertical Ge-on-Si APD	55.7	19.5	20 nA	26.5	13.5	Yes	1550	[56]
Ge/Si APD	12	0.9 (M = 1)	3 μA (M = 12)	28.5	7.5	N/A	1550	[57]
Ge-on-Si APD	101	0.41 (M = 1)	5.7 μA	55	30	N/A	1550	[58]
GeSn-on-Si APD	154	14.7	1.5 μA	21.1	13.1	N/A	1550	[59]
Vertical SACM Ge-on-Si APD	21.7	4.16	150 nA	soft breakdown	>48	Yes	1550	This work

The “N/A” means that the parameter is not provided by the paper.

## Data Availability

The data presented in this study are available on request from the corresponding author due to restrictions related to participant confidentiality.

## Supplementary Materials

The following supporting information can be downloaded at: https://www.mdpi.com/article/10.3390/mi17060726/s1.

## References

[1] Liu J. (2014). Monolithically Integrated Ge-on-Si Active Photonics. Photonics.

[2] Xue Y., Han Y., Tong Y., Yan Z., Wang Y., Zhang Z., Tsang H.K., Lau K.M. (2021). High-performance III-V photodetectors on a monolithic InP/SOI platform. Optica.

[3] Kang Y., Huang Z., Saado Y., Campbell J., Pauchard A., Bowers J., Paniccia M.J. (2011). High performance Ge/Si avalanche photodiodes development in intel. Proceedings of the 2011 Optical Fiber Communication Conference and Exposition and the National Fiber Optic Engineers Conference, 6–10 March 2011.

[4] Chen H.T., Verheyen P., Rakowski M., Heyn P.D., Lepage G., Coster J.D., Absil P., Roelkens G., Campenhout J.V. (2014). Low-voltage Ge avalanche photodetector for highly sensitive 10Gb/s Si photonics receivers. Proceedings of the 11th International Conference on Group IV Photonics (GFP), 27–29 August 2014.

[5] Hong C., Shi B., Qi F., Cai P., Duan Y., Hou G., Su T., Chiu T., Li S., Chen W. (2022). High speed Ge/Si avalanche photodiode with high sensitivity for 50Gbit/s and 100Gbit/s optical access systems. Proceedings of the 2022 Optical Fiber Communications Conference and Exhibition (OFC), 6–10 March 2022.

[6] Sakib M., Sun J., Kumar R., Driscoll J., Yeung K., Rong H. (2018). Demonstration of a 50 Gb/s all-silicon waveguide photodetector for photonic integration. Proceedings of the 2018 Conference on Lasers and Electro-Optics (CLEO), 13–18 May 2018.

[7] Orcutt J.S., Gill D.M., Proesel J., Ellis-Monaghan J., Horst F., Barwicz T., Xiong C., Anderson F.G., Agrawal A., Martin Y. (2016). Monolithic silicon photonics at 25 Gb/s. Proceedings of the 2016 Optical Fiber Communications Conference and Exhibition (OFC), 20–24 March 2016.

[8] Monroe D. (2016). Silicon photonics: Ready to go the distance?. Commun. ACM.

[9] Yoshida S., Shimada S., Matsuzaki A., Loo S.M., Otake Y., Nagayama T., Ochiai S., Tange T., Sasaki K., Yasui A. (2025). 10 μm Pitch Ge-on-Si SPAD Pixel Array with PDE of 33.8% at 1300 nm and 23.3% at 1550 nm under Room Temperature Environment. Proceedings of the 2025 IEEE International Electron Devices Meeting (IEDM).

[10] Na N., Lu Y.C., Liu Y.H., Chen P.W., Lai Y.C., Lin Y.R., Lin C.C., Shia T., Cheng C.H., Chen S.L. (2024). Room temperature operation of germanium–silicon single-photon avalanche diode. Nature.

[11] Ye H., Yu J. (2014). Germanium epitaxy on silicon. Sci. Technol. Adv. Mater..

[12] Endres J., Daniš S., Bauer G. (2013). The misfit dislocation density profile in graded SiGe/Si(001) layers prepared at different temperatures. J. Phys. Condens. Matter.

[13] Langdo T.A., Leitz C.W., Currie M.T., Fitzgerald E.A., Lochtefeld A., Antoniadis D.A. (2000). High quality Ge on Si by epitaxial necking. Appl. Phys. Lett..

[14] Klinger S., Berroth M., Kaschel M., Oehme M., Kasper E. (2009). Ge-on-Si p-i-n Photodiodes with a 3-dB Bandwidth of 49 GHz. IEEE Photon. Technol. Lett..

[15] Kang Y., Liu H.-D., Morse M., Paniccia M.J., Zadka M., Litski S., Sarid G., Pauchard A., Kuo Y.-H., Chen H.-W. (2008). Monolithic germanium/silicon avalanche photodiodes with 340 GHz gain–bandwidth product. Nat. Photon..

[16] Koester S.J., Schow C.L., Schares L., Dehlinger G., Schaub J.D., Doany F.E., John R.A. (2007). Ge-on-SOI-Detector/Si-CMOS-Amplifier Receivers for High-Performance Optical-Communication Applications. J. Light. Technol..

[17] Michel J., Liu J., Kimerling L.C. (2010). High-performance Ge-on-Si photodetectors. Nat. Photon..

[18] Zhang Y., Yang S., Yang Y., Gould M., Ophir N., Lim A.E.-J., Lo G.-Q., Magill P., Bergman K., Baehr-Jones T. (2014). A high-responsivity photodetector absent metal-germanium direct contact. Opt. Express.

[19] Virot L., Crozat P., Fédéli J.-M., Hartmann J.-M., Marris-Morini D., Cassan E., Boeuf F., Vivien L. (2014). Germanium avalanche receiver for low power interconnects. Nat. Commun..

[20] Tseng C.-K., Chen W.-T., Chen K.-H., Liu H.-D., Kang Y., Na N., Lee M.-C.M. (2013). A self-assembled microbonded germanium/silicon heterojunction photodiode for 25 Gb/s high-speed optical interconnects. Sci. Rep..

[21] Miyasaka Y., Hiraki T., Okazaki K., Takeda K., Tsuchizawa T., Yamada K., Wada K., Ishikawa Y. (2016). Ge/graded-SiGe multiplication layers for low-voltage and low-noise Ge avalanche photodiodes on Si. Jpn. J. Appl. Phys..

[22] Cong H., Xue C., Zheng J., Yang F., Yu K., Liu Z., Zhang X., Cheng B., Wang Q. (2016). Silicon Based GeSn p-i-n Photodetector for SWIR Detection. IEEE Photon. J..

[23] Su S., Cheng B., Xue C., Wang W., Cao Q., Xue H., Hu W., Zhang G., Zuo Y., Wang Q. (2011). GeSn p-i-n photodetector for all telecommunication bands detection. Opt. Express.

[24] Chen H., Verheyen P., De Heyn P., Lepage G., De Coster J., Balakrishnan S., Absil P., Yao W., Shen L., Roelkens G. (2016). −1 V bias 67 GHz bandwidth Si-contacted germanium waveguide p-i-n photodetector for optical links at 56 Gbps and beyond. Opt. Express.

[25] Zhang D., Xue C., Cheng B., Su S., Liu Z., Zhang X., Zhang G., Li C., Wang Q. (2013). High-responsivity GeSn short-wave infrared p-i-n photodetectors. Appl. Phys. Lett..

[26] Shim J., Kang D.-H., Yoo G., Hong S.-T., Jung W.-S., Kuh B.J., Lee B., Shin D., Ha K., Kim G.S. (2014). Germanium p-i-n avalanche photodetector fabricated by point defect healing process. Opt. Lett..

[27] Deeb H., Khomyakova K., Kokhanenko A., Douhan R., Lozovoy K. (2023). Dependence of Ge/Si Avalanche Photodiode Performance on the Thickness and Doping Concentration of the Multiplication and Absorption Layers. Inorganics.

[28] Alam M.S., Rahman M.S., Islam M.R., Bhuiyan A.G., Yamada M. (2007). Refractive index, absorption coefficient, and photoelastic constant: Key parameters of InGaAs material relevant to InGaAs-based device performance. Proceedings of the 2007 IEEE 19th International Conference on Indium Phosphide & Related Materials, 14–18 May 2007.

[29] Li C., Xue C.-L., Li C.-B., Liu Z., Cheng B.-W., Wang Q.-M. (2013). High bandwidth surface-illuminated InGaAs/InP uni-travelling-carrier photodetector. Chin. Phys. B.

[30] Li C., Li B., Qin S., Su J., He X., Guo X. (2018). Effects of Interface States on Ge-On-SOI Photodiodes. IEEE J. Electron Devices Soc..

[31] Latargez C., Durlin Q., Vialle C., Le Cocq M., Schembri A., Hartmann J.-M., Crochemore R., Lima G., Grosse P., André L. (2025). Study of Ge-on-Si avalanche photodiodes for short-wave infrared applications. Semicond. Sci. Technol..

[32] Aull B. (2016). Geiger-Mode Avalanche Photodiode Arrays Integrated to All-Digital CMOS Circuits. Sensors.

[33] Kindt W., Van Zeijl H. (1998). Modelling and fabrication of Geiger mode avalanche photodiodes. IEEE Trans. Nucl. Sci..

[34] Renker D. (2006). Geiger-mode avalanche photodiodes, history, properties and problems. Nucl. Instrum. Methods Phys. Res. Sect. A Accel. Spectrometers Detect. Assoc. Equip..

[35] Vickers J.S., Ispasoiu R., Cotton D., Frank J., Lee B., Kasapi S. (2003). Time-resolved photon counting system based on a Geiger-mode InGaAs/InP APD and a solid immersion lens. Proceedings of the 16th Annual Meeting of the IEEE Lasers and Electro-Optics Society, LEOS 2003, 27–28 October 2003.

[36] Donnelly J., Duerr E., Mcintosh K., Dauler E., Oakley D., Groves S., Vineis C., Mahoney L., Molvar K., Hopman P. (2006). Design Considerations for 1.06-μm InGaAsP–InP Geiger-Mode Avalanche Photodiodes. IEEE J. Quantum Electron..

[37] Cova S., Ghioni M., Lacaita A., Samori C., Zappa F. (1996). Avalanche photodiodes and quenching circuits for single-photon detection. Appl. Opt..

[38] Eaglesham D.J., Cerullo M. (1990). Dislocation-free Stranski-Krastanow growth of Ge on Si(100). Phys. Rev. Lett..

[39] LeGoues F.K., Copel M., Tromp R.M. (1990). Microstructure and strain relief of Ge films grown layer by layer on Si(001). Phys. Rev. B.

[40] Forrest S.R., Leheny R.F., Nahory R.E., Pollack M.A. (1980). In0.53Ga0.47As photodiodes with dark current limited by generation-recombination and tunneling. Appl. Phys. Lett..

[41] Chen Z., Jie B.B., Sah C.-T. (2006). Effects of energy distribution of interface traps on recombination dc current-voltage line shape. J. Appl. Phys..

[42] Khaliq A., Zhou X., Chai H.-Y., Ali M., Wu H., Gassab O., Liu H., Xiao D., Yang X.-G., Du S. (2024). Illumination Induced Negative Differential Resistance in InGaAs Avalanche Photodiode. IEEE Access.

[43] Windischhofer P., Riegler W. (2022). Passive quenching, signal shapes, and space charge effects in SPADs and SiPMs. Nucl. Instrum. Methods Phys. Res. Sect. A Accel. Spectrometers Detect. Assoc. Equip..

[44] Lu J., Park Y., Rozgonyi G.A. (2008). Deep level transient spectroscopy and capacitance-voltage study of dislocations and associated defects in SiGe∕Si heterostructures. J. Appl. Phys..

[45] DiLello N.A., Johnstone D.K., Hoyt J.L. (2012). Characterization of dark current in Ge-on-Si photodiodes. J. Appl. Phys..

[46] Bulyarskiy S.V., Lakalin A.V., Saurov M.A., Gusarov G.G. (2020). The effect of vacancy-impurity complexes in silicon on the current–voltage characteristics of p–n junctions. J. Appl. Phys..

[47] Musibau S., Poumpouridis N., Tsiara A., Franco J., Berciano M., Van Campenhout J., De Wout I., Crees K. (2024). Degradation and Recovery Kinetics Study of Vertical and Lateral Ge-on-Si Photodetectors. Proceedings of the 2024 IEEE International Reliability Physics Symposium (IRPS).

[48] Rehman S., Kim H., Patil H., Kadam K.D., Sagar R.U.R., Aziz J., Um D., Khan M.F., Kim D. (2021). Current Rectification, Resistive Switching, and Stable NDR Effect in BaTiO_3_/CeO_2_ Heterostructure Devices. Adv. Electron. Mater..

[49] Jia L., Zheng W., Huang F. (2021). Observation of negative differential resistance in SiO_2_/Si heterostructures. Cell Rep. Phys. Sci..

[50] Elahi E., Ahsan U., Khan M.F., Aziz J., Chauhan P., Michałowski P.P., Chen Y., Eda G., Loula M., Sarkar K.J. (2025). Electrical Transport of Nb-Doped MoS _2_ Homojunction P–N Diode: Investigating NDR and Avalanche Effect. Small.

[51] Suleman M., Kim M., Rehmat A., Elahi E., Asim M., Riaz M., Kumar S., Jung J., Seo Y. (2025). Exploring Double NDR Modulation and UV-NIR Photodetection in MoS_2_/Sb_2_Se_3_ Heterostructures. Adv. Opt. Mater..

[52] Irie K., McKinnon A.E., Unsworth K., Woodhead I.M. (2008). A Technique for Evaluation of CCD Video-Camera Noise. IEEE Trans. Circuits Syst. Video Technol..

[53] Li Y., Liu X., Li X., Zhang L., Li Y., Chen B., Zhi Z., Gao F., Li X., Guo P. (2022). Lateral separate absorption charge multiplication Ge-on-Si avalanche photodiode with low dark current in linear mode. Opt. Commun..

[54] Duan N., Liow T.Y., Lim A.E., Ding L., Lo G. (2012). 310 GHz gain-bandwidth product Ge/Si avalanche photodetector for 1550 nm light detection. Opt. Express.

[55] Yuan Y., Jung D., Sun K., Zheng J., Jones A.H., Bowers J.E., Campbell J.C. (2019). III-V on silicon avalanche photodiodes by heteroepitaxy. Opt. Lett..

[56] Kim G., Kim S., Kim S.A., Oh J.H., Jang K.S. (2018). NDR-effect vertical-illumination-type Ge-on-Si avalanche photodetector. Opt. Lett..

[57] Huang M., Li S., Cai P., Hou G., Su T.-I., Chen W., Hong C.-Y., Pan D. (2017). Germanium on Silicon Avalanche Photodiode. IEEE J. Sel. Top. Quantum Electron..

[58] Fleming F., Yi X., Mirza M.M.A., Jin X., Kirdoda J., Dumas D.C.S., Saalbach L., Modak M., Muir D.A.S., Smith C. (2024). Surface-normal illuminated pseudo-planar Ge-on-Si avalanche photodiodes with high gain and low noise. Opt. Express.

[59] Wanitzek M., Ramachandra H., Spieth C., Daus A., Schulze J., Oehme M. (2025). GeSn-on-Si Avalanche Photodiodes with High Responsivity and Low Dark Current. Adv. Electron. Mater..

[60] Wang Z., Jiang X., Meng W., Guo M., Huang Z., Liu G., Zhou J., Ke S. (2025). High-Gain Ge/Si Avalanche Photodetector with Stable Operation Temperature Up to 500 K. IEEE Electron Device Lett..

[61] Yi Q., Hu L., Huang B., Wang J., Wang T., Long T., Jiang W., Han T., Gu X., Guan Y. (2025). Novel Silicon Epitaxy-Based Avalanche Photodetectors with Parameter-Sensitivity Engineering for Enhanced Fabrication Robustness. IEEE Trans. Electron Devices.

