# Supplementary material for: Linear Multiplication Beyond Geiger Mode Threshold in Ge-on-Si Avalanche Photodiode
Source: Micromachines (Basel). 2026 Jun 15;17(6):726. doi: 10.3390/mi17060726 (PMC13304103; doi:10.3390/mi17060726)
Supplement: Supplementary file 1 [file micromachines-17-00726-s001.zip › micromachines-4284360-supplementary.pdf]

Support Information

Linear Multiplication Beyond Geiger Mode Threshold in Ge-on-Si Avalanche Photodiode

Dongyan Zhao [1,2,*], Qiang Wen [3], Fang Liu [1], Wei Qi [3], and Sichao Du [3,4,*]

1 Beijing Smart-Chip Microelectronics Technology Company Ltd., Beijing 100192, China; liufang@sgchip.sgcc.com.cn (F.L.)
2 College of Integrated Circuits, Zhejiang University (ZJU), Hangzhou 311200, China
3 Zhejiang Key Laboratory of Quantum Materials and Control, Zhejiang Engineering Research Center for Edge Intelligence Technologies and Equipment, School of Information and Electrical Engineering, Hangzhou City University, Hangzhou 310015, China; 2240201029@stu.hzcu.edu.cn (Q.W.); qiw@hzcu.edu.cn (W.Q.)
4 Zhejiang Province Key Lab of Intelligent Electromagnetic Control and Electronic Integration, Innovative Institute of Electromagnetic Information and Electronic Integration, College of Information Science and Electronic Engineering, Zhejiang University (ZJU), Hangzhou 310027, China
* Correspondence: dongyan-zhao@sgchip.sgcc.com.cn (D.Z.); sichaodu@zju.edu.cn (S.D.)

Section 1. Characteristics of the Ge-on-Si avalanche photodetector under high-bias conditions.

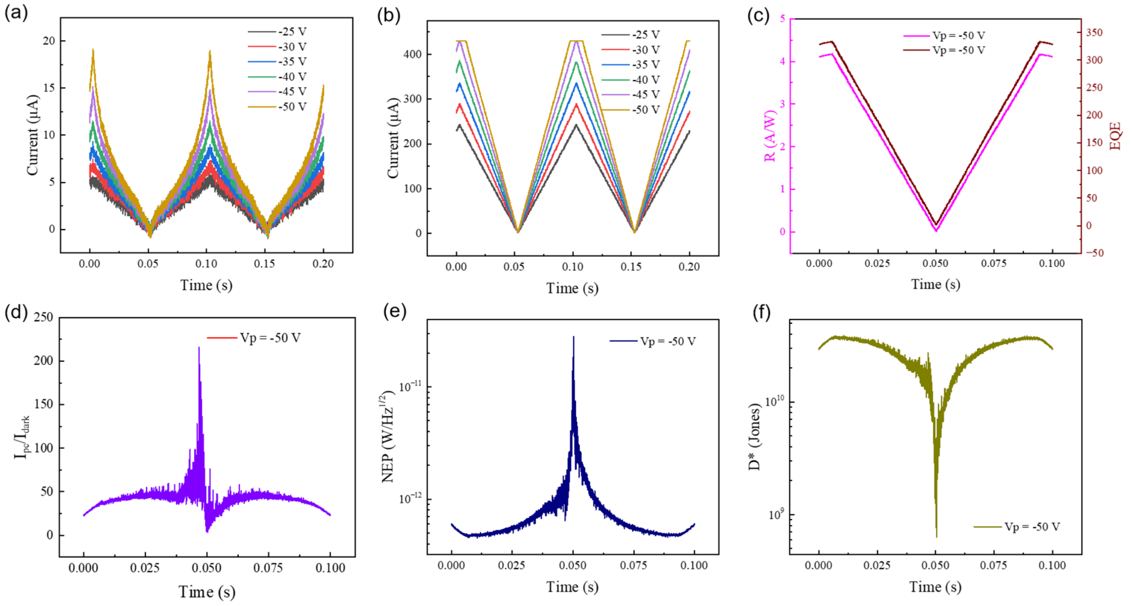

Figure S1. Photoresponse Characteristics of the Device under Periodic Ramp Gate Bias (Maximum Reverse Bias from 25 V to 50 V). (a) Current-time (*I-T*) characteristic curve of the device under dark conditions; (b) Photocurrent-time characteristic curve of the device under continuous illumination at 1550 nm wavelength and 100 μW power; (c) Variation of the extracted transient responsivity (*R*) and external quantum efficiency (*EQE*) with scanning time; (d) Transient characteristics of photocurrent-to-dark-current ratio (I_{pc}/I_{dark}); (e) Equivalent noise power (*NEP*) and (f) specific detectivity (D^*) as functions of time.

Figures S1 (a) and (b) depict the transient current-time (*I-T*) characteristics of the device under periodic ramp gate bias. The peak dark current of the device is approximately 19 μA, while the peak photocurrent under illumination reaches about 430 μA. Near the maximum peak bias, a distinct

saturation phenomenon appears at the crest of the photocurrent curve. Under the ramp gating voltage, for a device with a 1 μm-thick multiplication layer, the dark current exhibits a steepening slope at approximately −25 V, indicating that the device undergoes more intense impact ionization within this voltage range. However, the device does not show a sharp current surge; instead, it displays a gradual "soft breakdown" characteristic. This behavior is primarily attributed to the thicker multiplication layer, which exacerbates the dead-space effect. Additionally, during the dynamic sweep process, the space-charge effect induced by photogenerated carriers screens and weakens the effective electric field within the multiplication region, thereby suppressing the avalanche multiplication performance of the device.

The responsivity (*R*) and external quantum efficiency (*EQE*) extracted through dynamic scanning are shown in Figure S1 (c). As the bias voltage increases, the responsivity increases significantly, reaching a peak value of 4.16 A/W, with a corresponding *EQE* increase to 330%. The *EQE* substantially exceeding 100% provides direct evidence of carrier impact ionization and avalanche multiplication processes occurring within the device. As shown in Figure S1 (d), in the region near the peak bias, the photocurrent-to-dark-current ratio stabilizes within the range of 25 to 50 (the characteristic spikes during low-voltage switching transients arise from the dark current sharply dropping to the system's noise floor). Benefiting from the amplification of photogenerated carriers through avalanche multiplication, both the *NEP* and D^* of the device achieve optimal performance at the maximum bias (Figures S1 (e, f)). The *NEP* of the device decreases to a minimum of approximately 4.4×10^{-13} W/$\sqrt{\text{Hz}}$, while the specific detectivity reaches a maximum of about 4×10^{10} Jones.

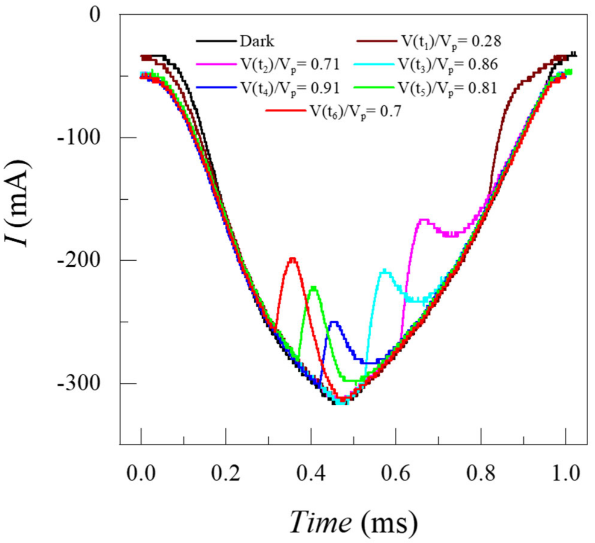

Figure S2. Dynamic current-time (*I-T*) response characteristics of a vertically structured SACM (Separate Absorption, Charge, and Multiplication) Ge-on-Si APD under ramp gating with synchronized pulsed illumination.

The figure S2 records the current variation of the device under a single-cycle (1 ms) ramp gating signal, tested at room temperature (uncooled). The superimposed ramp gating signal features a linear sweep with a peak voltage V_p = -80 V, synchronized with a 1550 nm pulsed laser (10 ns pulse width, 1 kHz repetition rate). The laser incidents occur at six characteristic instantaneous voltages $V(t_i)$, corresponding to bias voltages $V(t_i)$ = -(22.4, 56.8, 68.8, 72.8, 64.8, 56) V. Colored curves represent the responses under the respective illumination states, while the black curve denotes the dark response.

Key observations include: the dark current exhibits continuous linear growth with increasing reverse bias, without the current runaway characteristic of conventional Geiger mode. This behavior is attributed to the regulation of avalanche processes by dislocation traps at the Ge/Si interface and deep-level defects in the Si multiplication region. After laser triggering, all illumination-state curves display

a "two-stage transient" process: first a rapid current decay (attributed to the capture of photogenerated carriers by interface/deep-level traps), followed by a prolonged inverse quenching recovery (resulting from thermal emission of the trapped carriers).

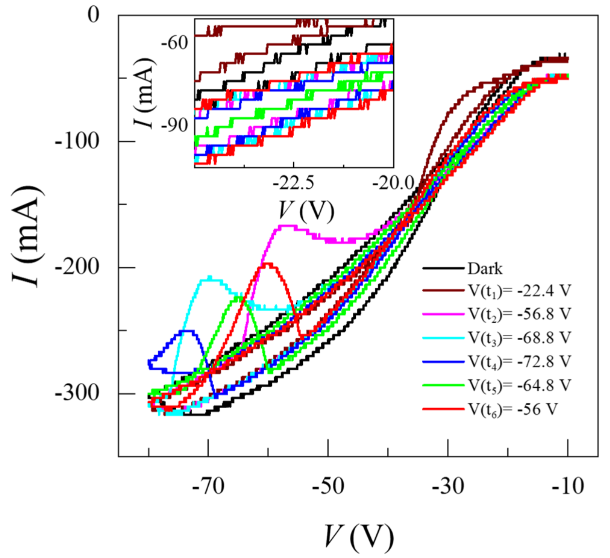

Figure S3. Current-voltage (*I-V*) characteristics of the vertical SACM (Separate Absorption, Charge, and Multiplication) Ge-on-Si avalanche photodetector.

The data in this graph are derived from the I-t curves of Figure S2, plotted by extracting the current values at each instantaneous voltage $V(t_i)$ during the forward scan (ramp-up) and reverse scan (ramp-down) processes. The testing conditions are consistent with those of Figure S2. The colored curves represent the phototransient responses at different $V(t_i)$, while the black curve represents the dark response. The inset shows an enlarged view of the low-bias region.

Key features in the graph include: First, a significant *I-V* hysteresis effect—where the forward and reverse scan curves exhibit a clear offset, with a maximum current difference of up to 50 μA in the high-bias region. This is attributed to charge trapping at the Ge/Si interface, carrier capture by deep-level traps in the Si multiplication region, and dopant interdiffusion (non-abrupt junction) caused by thermal processing. Second, the high-bias region exhibits negative differential resistance, characterized by a decrease in current as the reverse bias increases. This results from the combined effects of light-induced electric field self-quenching in the Si multiplication region (where photogenerated carriers neutralize space charges) and modification of the Ge/Si interface barrier (where trap capture of carriers reduces the barrier height). This graph confirms the role of traps in modulating carrier transport and verifies the reverse modulation capability of light on the device.

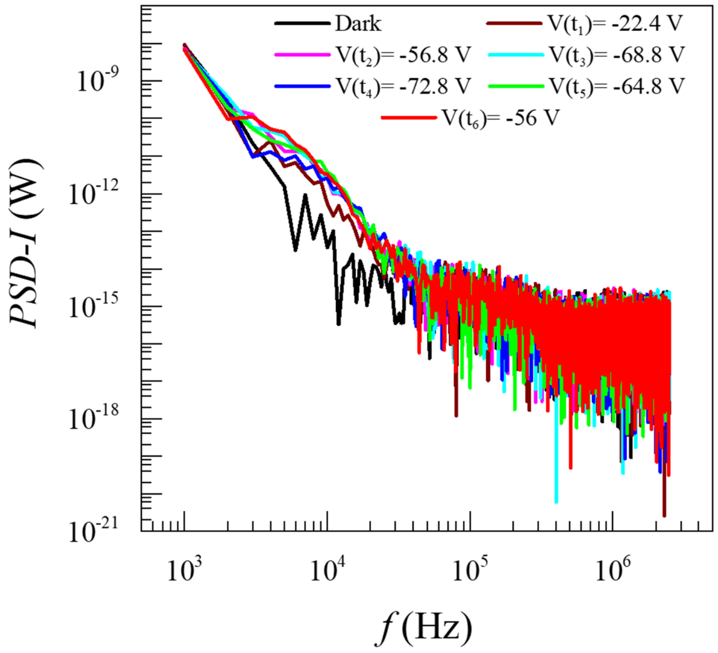

Figure S4. Power spectral density (PSD) calculated over one complete ramp gating cycle for dark- and various illuminated states by a 1550 nm pulsed laser, corresponding to $V(t_i)$ values of -(22.4, 56.8, 68.8, 72.8, 64.8, 56) V.

Figure S4 shows the power spectral density (PSD) distribution of a Ge-on-Si avalanche photodiode under single-cycle ramp gating. Through fast Fourier transform (FFT) processing of the time-domain current signals obtained from Figure S2, spectral responses were acquired under dark conditions and at different normalized instantaneous gating voltages $V(t_i)$ = -(22.4, 56.8, 68.8, 72.8, 64.8, 56) V. The results reveal significant differences in the low-frequency region, primarily caused by thermal noise and dark current fluctuations. Under illumination, the PSD curves exhibit varying degrees of fluctuation with changing gating voltages, reflecting the modulation of low-frequency noise through photoinduced trap filling/release processes and electric field redistribution. In the mid-to-high frequency range, condition-independent and frequency-stable PSD is observed, indicating the dominance of readout circuit noise components.

Section 2. Photoresponse characteristics of the Ge-on-Si photodetector under low-bias conditions.

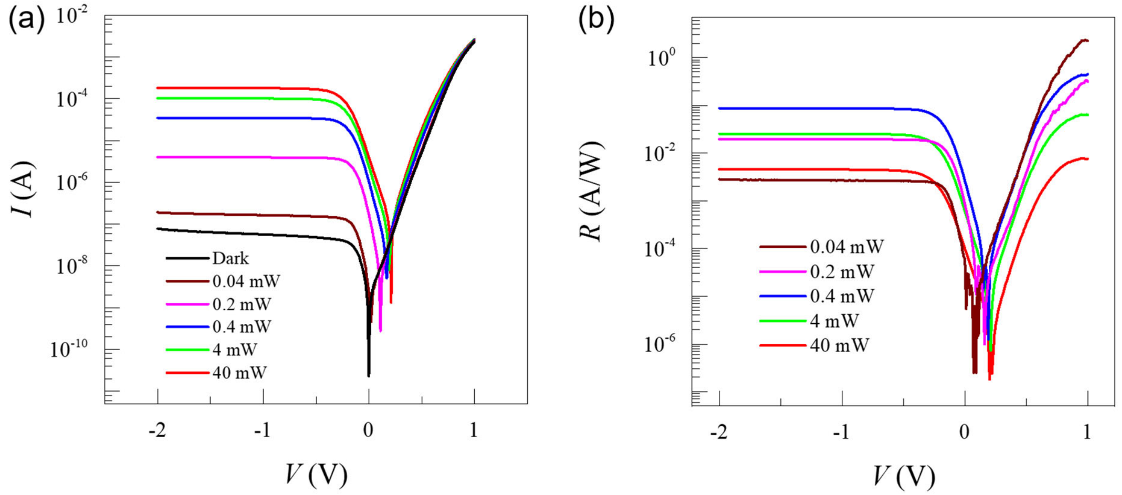

Figure S5. (a) I-V characteristics of the device under dark state and different incident power levels (0.04, 0.2, 0.4, 4, and 40 mW) with voltage swept linearly from -2 V to +1 V. (b) Responsivity (R) as a function of bias voltage V evaluated at unmodulated optical power levels of 0.04, 0.2, 0.4, 4, and 40 mW.

In this section, we present the dark and photo response characteristics of the Ge-on-Si APD under low bias, as demonstrated through the I-V measurements under continuous illumination with 1550 nm light. The voltage V is swept linearly from −2 V to 1 V. A photovoltaic open-circuit voltage (V_{oc}), commonly observed in photodiodes and enabling their potential use in solar cells, is produced as shown in Figure S5. For the Ge-on-Si APD characterized by Wm = 1μm, the punch-through voltage is reduced to approximately (−0.15, −0.25) V. Another interesting deduction is the distinguishable photo response in the forward bias domain. The forward operational resistance for respective APD at 1 V under dark condition is 42.6 kΩ.

Responsivity (R) which is a metric relating photocurrent (I_{pc}) to the incident illumination intensity (P) through $R = I_{pc}/P_{in}$ is shown in Figure S5. In general, the condition of R>1 indicates sufficient carrier amplification. However, in the low-bias testing conditions, the electric fields established in the multiplication region is below the avalanching threshold of approximately 1.5×10^7 V/m. The highest responsivity R of 0.087 *A*/*W* was achieved at an incident illumination intensity of 0.4 mW, which meets the requirements for the best signal-to-noise ratio (*SNR*). At 4 mW and 40 mW illuminations, R tends to reduce due to saturation effects. Whereas, at 0.04 mW and 0.2 mW illuminations, relatively low SNR imposes smaller R values. The optimal R values are obtained at an illumination intensity that provides the best SNR coupled with minimal recombination after the carriers are released from their traps.

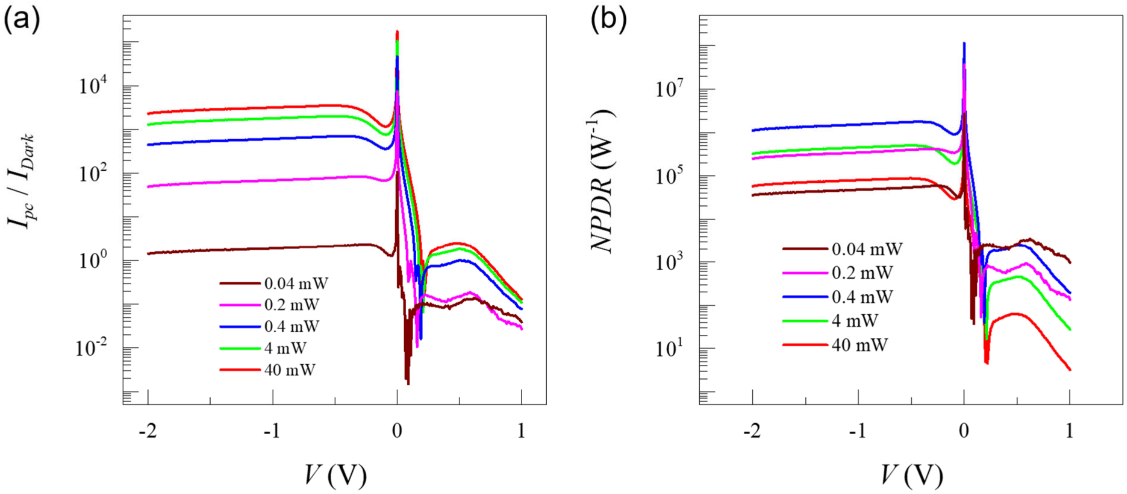

Figure S6. (**a**) The ratio of photocurrent to dark current (I_{pc}/I_{dark}) and (**b**) the normalized photo-to-dark current ratio (NPDR) as functions of V under unmodulated incident light intensities of 0.04, 0.2, 0.4, 4, and 40 μW.

Figure S6(a) demonstrates the device's capability to amplify photoresponse relative to dark noise: at low optical power levels, the ratio increases sharply with rising optical power, indicating excellent sensitivity and detection capability for weak optical signals; while it exhibits a saturation tendency under high optical power. The capability of the Ge-on-Si APD to perform as an illumination-controlled switch is presented in Figure S6 (a), where photo-to-dark current ratio (I_{pc}/I_{dark}) is evaluated. In the reverse biased domain for device having the maximum I_{pc}/I_{dark} under (0.04, 0.2, 0.4, 4, 40) mW illuminations are (2.29, 83, 707, 2011, 3510), corresponding to reverse biases of −(0.2, 0.28, 0.41, 0.49, 0.54) V. Figure S6 (b) shows another figure of merit, namely, normalized photo-to-dark-current ratio (*NPDR*), which is evaluated by normalizing I_{pc}/I_{dark} with reference to input unmodulated illumination intensity P. The *NPDR* can assist in comparing R, and external quantum efficiency of different detectors for a reference I_{dark}.

If Figure S6 (a) and S6 (b) are compared carefully, the *NPDR* signatures for the device with W_m = 1000 nm under P = (0.04, 0.2, 0.4, 4, 40) mW are signature-wise resembling as *NPDR* results from the scaling of I_{pc}/I_{dark} through P or translation of R via I_{dark} as shown below,

$$NPDR = I_{pc}/I_{dark}/P = I_{pc}/P/I_{dark} = R/I_{dark} \quad (1)$$

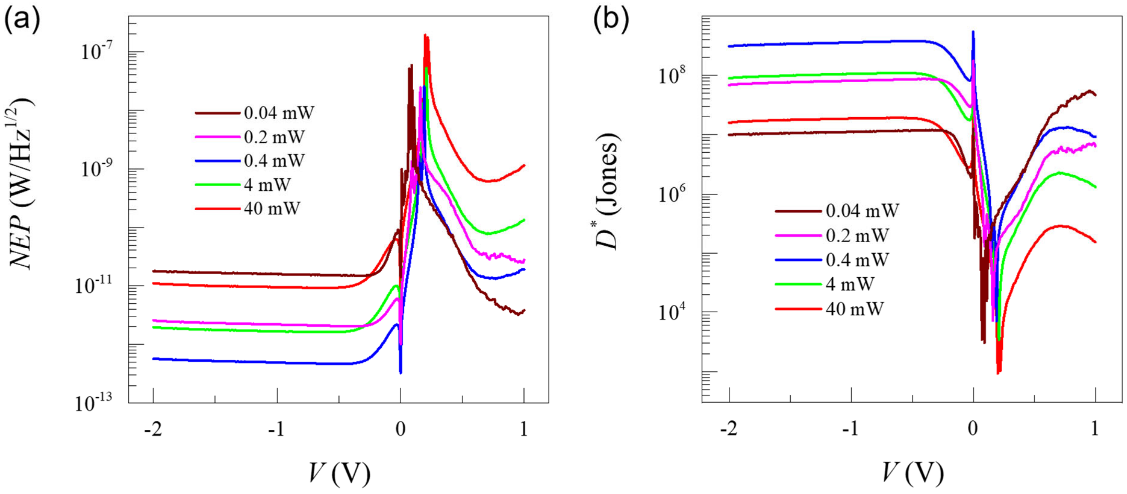

Figure S7. (**a**) The noise-equivalent-power (*NEP*) and (**b**) specific detectivity (D^*) versus *V* under unmodulated incident light intensities of 0.04, 0.2, 0.4, 4, and 40 μW.

Furthermore, we plot the noise-equivalent-power (*NEP*), which is a metric for evaluating optical power with *SNR* = 1. From data demonstrated in Figure S7 (a), we accomplish that Ge-on-Si APD perform noticeably well at 0.4 mW illumination resulting in lowest *NEP* values. The relatively positive slopes in the reverse biased result from the inverse correlation of *NEP* with I_{pc}/I_{dark} and *NPDR*. The largest *NEP* values correspond to the weakest illumination 0.4 mW offering lowest *SNR*. The worst *NEP* values are direct consequence of compromised drift during charge transport. For a certain APD having an area (*A*) and characterized with I_{dark}, the specific detectivity (D^*) is estimated using $D^* = R\sqrt{A}/\sqrt{2eI_{\mathrm{dark}}}$ and subsequently plotted in Figure S7 (b). D^* is basically $NPDR/\sqrt{AI_{\mathrm{dark}}/2e}$. And it has a sort of direct relationship with other figures of merits such as *R* and I_{pc}/I_{dark}. At large illumination intensities, D^* is reduced due to saturation phenomena, while under smaller illumination intensities, *SNR* become insignificant.
